# Examining the role of memory in social cognition

**DOI:** 10.3389/fpsyg.2013.00437

**Published:** 2013-07-12

**Authors:** R. Nathan Spreng

**Affiliations:** Laboratory of Brain and Cognition, Department of Human Development, Cornell UniversityIthaca, NY, USA

The function of memory is not only to recall the past, but also to form and update models of our experiences and use these models to navigate the world. Perhaps, the most complex environment for humans to navigate is the social one. Social dynamics are extraordinarily complex, unstructured, labile and difficult to predict. Successful navigation through our many social landscapes is essential to forming and maintaining the durable social bonds necessary for physical and mental health. Until recently, little research has examined the role that memory plays in social behavior and interpersonal sensitivity. There is growing evidence that recalling personally experienced events (autobiographical memory) and inferring the mental states of others (mentalizing or theory-of-mind) share an extensive functional neuroanatomy (Buckner and Carroll, 2007; Spreng et al., 2009; Spreng and Grady, 2010; Rabin et al., 2010) and may be critical for adaptive social cognition. The functional overlap between autobiographical memory and mental inference has been hypothesized to facilitate the integration of personal and interpersonal information (Spreng and Mar, 2012). This integration may provide a means for personal experiences to become social conceptual knowledge that, in turn, informs strategic social behavior. In this process, we project our memories onto others in order to better understand and empathize with their experiences (Perry et al., 2011).

The contributions to this Research Topic have examined this intersection between cognitive and social neuroscience, exploring the importance of memory to social cognition. What has become increasingly clear is that memory interacts with social cognitive processes in a diversity of meaningful and interesting ways. This interaction is reflected here through a range of individual differences, neuropsychological and developmental studies. The convergence of findings across the papers is very encouraging and provides good support for the central proposition that memory is a significant contributor to adaptive social cognition. The emergent themes are briefly reviewed here along with a few concluding remarks on future research directions.

The constructive nature of memory, whereby elements of a prior experience are woven back together during recollection, also supports imagination, whereby elements of disparate prior experiences are woven together in novel ways (Schacter et al., 2012). In this way, the network of brain regions supporting the recollection of prior experiences also allows one to imagine the experiences of other people (Hassabis et al., 2013). Gaesser (2012) proposes that this process may facilitate empathy and promote prosocial behavior. The constructive nature of memory also leaves recollection subject to distortions (Schacter, 2012). Brown et al. (2012) argue that this malleable nature of memory serves a socially adaptive function: The content of personal memories merge in the process of social interaction and this, in turn, fosters a sense of collective identity. The work of Lindner et al. (2012) suggests that there may be certain boundaries to this effect, however, that are driven by group membership.

In navigating the social world, we must often retrieve, maintain, manipulate, and update the information we have about other people. Meyer and Lieberman (2012) review the literature examining the unique neural dynamics underlying social working memory. In an empirical paper, Artuso et al. (2012) explore factors that impact working memory updating for faces. Cassidy and Gutchess (2012) provide preliminary evidence for a social memory system, potentially dissociable from the hippocampal declarative memory system. They found that amygdala and ventromedial prefrontal cortex integrity in older adults was associated with the successful retrieval of impressions for others (2012).

When one considers the broad range of interpersonal interactions encountered on a daily basis, it becomes clear that there are substantial differences in how people are able to navigate social situations. Some of these differences may relate to how we use memory in a social context. Ciaramelli et al. (2013) found that memory for previous episodes modulates the empathic response to others in a similar situation. Yang et al. (2012) found that the propensity for one to spontaneously relate one's personal memories to that of another individual predicted medial temporal lobe connectivity measured during resting-state fMRI.

It is becoming increasingly clear that the loss of a record of the personal past in neurological populations impairs the ability to adaptively navigate the social world. Four research articles converged around this central point with potentially intriguing implications for clinical practice. Davidson et al. (2012) reported that patients with amnesia are less likely to form and maintain social bonds, and have a smaller social network size. Adult onset hippocampal amnesia patients reported lower levels of trait empathy and demonstrated no increase in prosocial behavior with an empathic mood induction (Beadle et al., 2013). Consistent with recent neuroimaging findings demonstrating that the hippocampus is engaged during theory of mind of personally familiar individuals but not unfamiliar ones (Rabin and Rosenbaum, 2012), a patient with developmental amnesia was specifically impaired in providing a rich description of events related to oneself and close others, but not unfamiliar others (Rabin et al., 2012). Also examining developmental amnesia resulting from perinatal hypoxia, Staniloiu et al. (2013) found impairments on complex social judgment and perception tasks. Similar findings have been observed in neuropsychiatric disorders that are known to impair social cognition. In a longitudinal case study charting the development of a child with autism spectrum disorder, Bon et al. (2012) present an evolving picture of atypical autobiographical memory functioning. In their review, Dimaggio et al. (2012) report that shared impairments in autobiographical memory and mentalizing are present in both psychiatric and personality disorders and these deficits need to be considered in treatment and intervention protocols.

Memory and social cognition are diverse concepts. This Research Topic reveals multiple points of convergence, from imaginative and empathic experience to the idea of a discrete social memory system. The importance of memory to social cognition also emerged in the context of memory disorders with important rehabilitation implications. As a collection, these works have begun to explore how our past experiences come to bear on our ability to navigate the social world, yet a number of fundamental questions remain. How are social experiences distilled into meaningful and flexible representations? By what mechanism is social conceptual knowledge represented, updated, and used to guide social behavior? What is unique about the neural coding of social information in memory? How do these sociomnemonic processes relate to the functions of the default network and other large-scale brain network interactions? Delineating these relationships will reveal how we use a record of the past to successfully navigate through our many and varied social landscapes.
